# High rate of extra-haematological toxicity compromises dose-dense sequential adjuvant chemotherapy for breast cancer

**DOI:** 10.1038/bjc.2011.414

**Published:** 2011-10-18

**Authors:** E Brain, C Levy, D Serin, H Roché, M Spielmann, R Delva, C Veyret, L Mauriac, M Rios, A L Martin, M Jimenez, B Asselain, M Gauthier, F Bonnetain, P Fumoleau

**Affiliations:** 1 Department of Medical Oncology, Institut Curie – Hôpital René Huguenin, 35 rue Dailly, 92210 Saint-Cloud, France; 2 Breast Oncology Unit, Centre de Lutte Contre le Cancer – Centre François Baclesse 3, Avenue Général Harris, 14076 CAEN cedex 05, France; 3 Breast Oncology Unit, Institut Sainte-Catherine, 1750 Chemin du Lavarin, 84000 Avignon, France; 4 Department of Medical Oncology, Institut Claudius Regaud, 20-24, rue du Pont-Saint-Pierre, 31052 Toulouse cedex, France; 5 Department of Medical Oncology, Institut de Cancérologie Gustave Roussy, 114 rue Edouard Vaillant, 94805 Villejuif, France; 6 Breast Oncology Unit, Centre Paul Papin, 2 rue Moll, 49000 Angers, France; 7 Department of Medical Oncology, Centre Henri Becquerel, rue d’Amiens, 76038 Rouen cedex 1, France; 8 Department of Medical Oncology, Centre Régional de Lutte Contre le Cancer – Institut Bergonié, 229 cours de l’Argonne, 33076 Bordeaux cedex, France; 9 Department of Medical Oncology, Centre Alexis Vautrin, avenue de Bourgogne, 54511 Vandoeuvre-les-Nancy, France; 10 UNICANCER – Fédération Nationale des Centres de Lutte Contre le Cancer, 101, rue de Tolbiac, 75654 Paris, France; 11 Department of Bioinformatics – Institut Curie, 26 rue d’Ulm, 75248 Paris, France; 12 Centre Georges François Leclerc, 1 rue Professeur Marion, 21000 Dijon, France

**Keywords:** adjuvant treatment, docetaxel, dose-dense regimen, early breast cancer, skin toxicity, taxanes

## Abstract

**Background:**

A dose-dense strategy has been considered to improve results of adjuvant chemotherapy for breast cancer. This randomised phase II trial investigated the feasibility of this approach with sequential anthracyclines and taxanes-based chemotherapy.

**Methods:**

Patients with high-risk node-positive breast cancer were treated with three cycles of fluorouracil 500 mg m^−2^, epirubicin 100 mg m^−2^, cyclophosphamide 500 mg m^−2^ (FEC 100) followed by three cycles of docetaxel 100 mg m^−2^ delivered at 2-weekly intervals supported by primary prophylaxis with filgrastim. All patients were randomised to either uninterrupted treatment (arm A) or to have a 2-week additional period of rest between the FEC and docetaxel (arm B). The primary endpoint was the rate of success of chemotherapy delivery. Using a two-stage Fleming design, 120 patients were required with one interim analysis.

**Results:**

In March 2005, enrolment was stopped into arm A after the observation of severe skin toxicities. Following the planned interim analysis, the study was closed because of the high rate of grade 3/4 skin toxicities in both arms (arm A: 32.4% and arm B: 18.9%).

**Conclusion:**

Sequential dose-dense FEC 100 followed by docetaxel 100 mg m^−2^ is not feasible. Feasibility still depends largely on several factors including the choice of drugs, dosage and sequence of administration.

Over the last three decades, adjuvant systemic chemotherapy for early breast cancer has delivered steadily improving outcomes, as a result of the introduction of new cytotoxic agents such as anthracyclines and taxanes, the optimisation of standard regimens administration, and more recently, better patient selection using elaborate prognostic algorithms (Dang and Hudis, 2006; Levine and Whelan, 2006). Polychemotherapy regimens based on an anthracycline and taxane backbone have emerged as the international standard in high-risk breast cancer including node positive (pN+) cases (Goldhirsch *et al*, 2009). Further attempts to improve the outcomes with standard chemotherapy have focused on either dose intense (higher dose) or dose-dense (more frequent administration) strategies, both being made possible with highly effective granulocyte colony-stimulating factors (G-CSF). For the dose-dense strategy, models of tumour growth and response based on the Norton–Simon hypothesis (Norton *et al*, 1976) were translated into regimens, which aimed to increase tumour cell kill by decreasing the time intervals between treatments, preventing cancer cell repopulation, particularly in tumours with high proliferation rates. This strategy was fully evaluated in the Cancer and Leukemia Group B 9741 adjuvant trial, which demonstrated significant benefits compared with the conventionally scheduled four cycles of standard doxorubicin and cyclophosphamide (AC) followed by four cycles of paclitaxel (Citron *et al*, 2003). However trans-Atlantic differences in the preferred anthracycline regimen hampered the further application of this approach to Europe where fluorouracil 500 mg m^−2^, epirubicin 100 mg m^−2^ and cyclophosphamide 500 mg m^−2^ regimen (FEC 100) (French Adjuvant Study Group, 2001) or the Canadian cyclophosphamide, epirubicin and flurouracil regimen (Levine *et al*, 1998) are more widely used than AC. Uncertainties also persist regarding the optimal choice of taxane and the best way to deliver it: docetaxel might be more active than paclitaxel based on results obtained in the metastatic setting, and concomitant schedules (Brain *et al*, 2005) might be responsible for more side effects than the genuinely sequential ones (Bear *et al*, 2003). In order to resolve these differences, we chose to investigate the feasibility of two dose-dense versions of the standard FEC-D sequential chemotherapy regimen consisting of three cycles of FEC 100 followed by three cycles of docetaxel 100 mg m^−2^ (D), as used on a 3-weekly basis in the PACS 01 adjuvant trial run by the Fédération Nationale des Centres de Lutte Contre le Cancer (UNICANCER) (Roché *et al*, 2006). The phase II study was designed to identify the optimal 2-weekly version of FEC-D, which could then be taken into a phase III trial against standard 3-weekly FEC-D.

## Materials and methods

### Patient population

Eligible patients were recruited from 14 French cancer centres. They were women, aged between 18 and 65 years with unilateral pT1–pT3-operated breast cancer, clear surgical margins and axillary node clearance including at least five lymph nodes. Main eligibility criterion was a ‘high-risk’ pN+ disease defined as either one, two or three positive nodes and negative oestrogen and progesterone receptors status (ER-negative and PgR-negative) or more than three positive nodes irrespective of the hormone receptor status. Main inclusion criteria included the following: WHO performance status <2; interval period between first surgery and start of adjuvant chemotherapy of less than 60 days; normal left ventricular ejection fraction; normal haematological, renal and liver functions. Patients were excluded in the case of any evidence of distant metastasis, documented history of previous cancer (except treated basal cell of the skin and uterine cervix cancer), cardiac disease, any chronic digestive disease, B or C hepatitis, and serious underlying medical or psychiatric illness. Pregnant or breast-feeding women were ineligible, and contraception was mandatory for those of child-bearing age.

Potentially eligible patients underwent bone scan, chest radiograph, abdominal ultrasound or computed tomography, and ultrasound or radionuclide cardiac scan before being randomly assigned to treatment after giving written informed consent.

### Treatment regimens

Patients were assigned to one of the two regimens by the central office of the UNICANCER Bureau des Etudes Cliniques et Thérapeutiques (Paris, France), which guaranteed allocation concealment. Randomisation was stratified according to the participating centre only, using a 1 : 1 computerised randomisation.

Both arms consisted of three cycles of FEC 100 followed by three cycles of docetaxel 100 mg m^−2^, given intravenously (1-h infusion) either every 2 weeks without interruption (arm A), or with a 2-week additional period of rest between the third cycle of FEC 100 and first cycle of docetaxel (arm B).

Daily G-CSF (filgrastim) support was mandatory (5 *μ*g kg^−1^ per day subcutaneously from day 3 to day 10) for each cycle, as well as standard docetaxel steroids-based premedication.

Toxicity evaluations using the National Cancer Institute Common Toxicity Criteria Version 3.0 were performed on day 1 of each cycle of chemotherapy.

In case of grade 2 neutropenia or grade 1 thrombocytopenia, the next cycle of chemotherapy was to be postponed by 1 or 2 weeks to allow patient recovery. The same guideline was applied in case of extra-haematological toxicity (excluding alopecia, nausea and vomiting) to allow recovery to a grade ⩽1. In both cases, treatment was to be stopped if recovery did not occur after a 2-week delay.

Dose reduction by 25% was to be applied in the event of febrile neutropenia (defined as a grade 4 neutropenia with temperature above 38.5°C having required antibiotics and/or having lasted more than 24 h), thrombocytopenia grade 4 or extra-haematological toxicity grade ⩾3 (excepting alopecia, nausea and vomiting). In the event of toxicity recurrence despite dose reduction, treatment was to be stopped.

External beam radiotherapy and endocrine treatment were to follow the last cycle of chemotherapy according to standard guidelines.

### Statistical design

The primary endpoint of this study was the rate of success of chemotherapy delivery in both arms, without dose reduction or treatment delay. A total of 120 patients were necessary to give 97% power according to a two-stage Fleming design with a two-sided type I error of 3%, allowing 30% (H0) of dose reduction compared with 11% (H1) in the q3w experimental arm (three FEC 100, three docetaxel) of the PACS 01 trial (Roché *et al*, 2006).

An intermediate analysis was to be performed after inclusion of 30 patients into each arm, without temporary stop of enrolment, before extending to a full recruitment of 60 patients into each arm only if less than eight patients per arm had required a dose reduction or cycle postponement for reasons of toxicity. At the final analysis, the observation in an arm of 12 or more patients requiring dose reduction or cycle delay would lead to the conclusion that such a dose-dense schedule was not feasible.

Secondary endpoints included safety profile, dose intensity (mg m^−2^ per week), relapse-free survival and overall survival.

Qualitative data were reported by frequency and 95% confidence interval, and compared using Pearson's *χ*^2^-test or Fisher's exact test. Quantitative data were summarised by mean, s.d., median and range values, and compared as appropriate with Student's *t*-test or the Mann–Withney or Wilcoxon tests. All the analyses were based on the intention-to treat principle and performed with the R software (version 2.0.1, http://cran.univ-lyon1.fr/, Lyon, France). All the tests were bilateral and a threshold of 5% or less was to be considered significant.

A steering committee was in charge of supervising the study and monitoring patient accrual, treatment compliance and safety. The protocol of this study (EudraCT No 2004-002031-11) was approved by the institutional review board of each participating centre and by the study ethics review committee. It was conducted according the Declaration of Helsinki and the Good Clinical Practice guidelines.

## Results

### Conduct of the study

**Figure 1:**
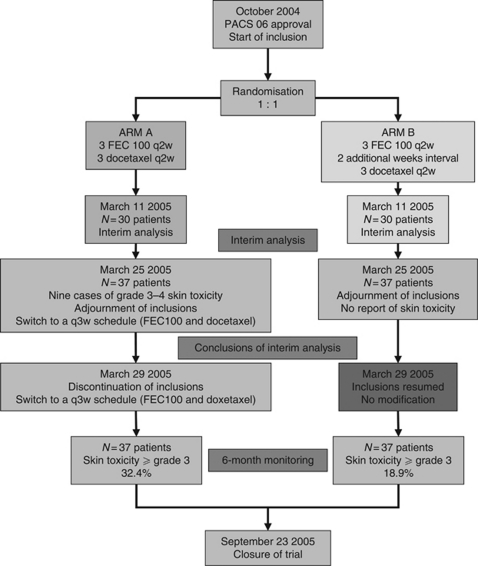
Study flow chart.

Enrolment started in October 2004. According to the statistical design, an interim analysis was to be conducted after enrolment of the sixtieth patient. However, before that, the identification of nine cases of grade ⩾3 skin toxicity occurring after the first cycle of docetaxel in arm A (leading to withdrawal from treatment in five patients) resulted in suspension of recruitment in March 2005 in both arms, pending full interim safety analysis. All patients in arm A were switched to a classical 3-week interval for the remaining cycles (FEC 100 or docetaxel). As no extra-haematological toxicity, grade ⩾3, had yet been reported in arm B, recruitment to arm B was allowed to resume, but in fact no further enrolment occurred, reflecting perhaps investigators’ concern as to the feasibility of this regimen as well. In September 2005, after completion of the interim analysis, the steering committee decided to terminate the study with 37 patients enrolled in each arm, because of the unexpectedly high rate of skin toxicity (grade ⩾3) in both arms (32.4% and 18.9% of patients in arm A and B, respectively, Figure 1).

### Patient population

Apart from a slight imbalance for age, type of surgery and nodal status favouring arm A, the patients’ characteristics were relatively well balanced between the two groups (Table 1). More than 50% of tumours were Scarf Bloom and Richardson grade III. The status of both ER and PgR was negative in 30% of cases whereas 27% of tumours showed HER2 overexpression.

### Tolerance

Table 2 summarises the distribution of main side effects according to arm and regimen component. For reasons of clarity, the data shown combine all events recorded in each arm until the trial closure of the trial (September 2005), including also those occurring in arm A after the shift to a 3-weekly schedule in that arm in March 2005.

Haematological toxicity did not differ between both arms, neutropenia occurring in one third of patients during FEC (cycle 1–3) with less than 6% of patients developing one or more febrile event during either sequential regimen. Neither grade 4 thrombocytopenia nor any grade 5 event was reported.

Extra-haematological toxicity peaked from cycle 4 (first cycle with docetaxel) in both arms, including skin and nail toxicity, arthralgia/myalgia and fluid retention.

Grade 3–4 skin toxicity occurred in 32.4% and 18.9% of patients in arm A and B respectively, all during the docetaxel component of therapy, with lower rates of grade 1–2 nail toxicity events, arthralgia/myalgia and grade 1–2 oedemas being seen in arm A. Of note, no grade 3–4 skin toxicity was observed in arm A after the intercycle interval was set to 3 weeks in March 2005 (data not shown).

**Figure 2:**
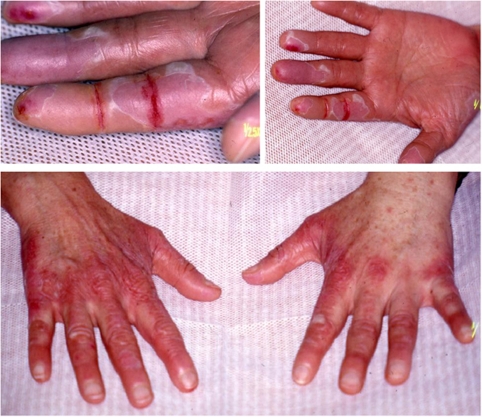
Photographs of grade 4 palmo-plantar erythrodysesthesia.

Skin toxicity included dermatitis, erythematous diffuse or peripheral eruptions, pruritus, labial and peripheral oedema, culminating in grade 4 palmo-plantar erythrodysesthesia (Figure 2). Most resolved, some with long delays exceeding 3 to 6 months, but some patients were left with persisting skin problems and head alopecia.

### Treatment delays and dose reductions

A total of 54 cycles were given on a 3-weekly basis following discontinuation of enrolment in arm A whereas 390 cycles were administered according to the allocated treatment schedule. When considering all cycles in each arm, significant delays (⩾5 days), dose reductions and cycle cancellations occurred in 66 (30%), 11 (5%) and 15 (7%) of cycles *vs* 15 (7%), 7 (3%) and 6 (3%) in arm A and arm B, respectively. Excluding the 54 cycles deliberately given on a 3-weekly schedule, the rate of significant delays in arm A lowered to 7% (12 out of 168 cycles) (see detail in Table 3). Except one cycle cancelled in arm B, one cycle with a 25% dose reduction in each arm and four delays at cycle 2 in arm A, there was no dose density alteration of FEC 100 administration in either arm. Most of the treatment modifications occurred from cycle 5 onwards, after the first cycle of docetaxel, and mostly due to toxicity. In total, nine patients stopped docetaxel in arm A compared with three in arm B.

## Discussion

In this study, administration of adjuvant, dose-dense FEC 100 therapy with G-CSF followed by dose-dense docetaxel with G-CSF proved to be not feasible in women with high-risk node-positive early-stage breast cancer. Whatever schedule chosen, with (arm B) or without (arm A) an extra 2-week interval between both sequences, dose-dense chemotherapy yielded an unacceptable high rate of grade 3–4 skin toxicity (32.4% and 18.9% in arm A and B, respectively). This led to the termination of the trial in two steps: (i) following the interim analysis (at 6 months of enrolment), early closure and shift from a continuous bi-weekly regimen to a continuous tri-weekly schedule for arm A; (ii) final closure of the trial after an additional 6-month careful monitoring, which showed a similar unacceptable toxicity profile in arm B.

Although the advent of neutrophil-stimulating growth factors has permitted exploration of the benefits of administering chemotherapy at shortened intervals (so-called ‘dose-density’) with manageable or even decreased haematological toxicity (Citron *et al*, 2003; Jones *et al*, 2009), it also revealed extra-haematological side effects unusual with the use of standard scheduling. In our trial, febrile neutropenia rates were low and we did not observe any other significant grade 3–4 haematological toxicity. Conversely, extra-haematological toxicity of grade 2 or even 1 was noted, which may prove to be significant. Thus not only was grade 3–4 skin toxicity increased but also grade 1–2 nail toxicity, fluid retention, and arthralgia/myalgia, some occurring in up to one third of patients. Of note, the incidence of all these extra-haematological side effects peaked during the docetaxel component right from cycle 4, and the lower global rates favouring arm A likely reflect the conservative measure to increase by 1 week the interval between each remaining cycle of chemotherapy in this group after March 2005 (Table 2).

Nevertheless, dose-dense chemotherapy for early-stage breast cancer remains an important strategy as it is predicted to maximise the impact of individual cytotoxic agents (Norton *et al*, 1976; Norton and Simon, 1977; Budman *et al*, 1998), with encouraging results on long-term outcome (Citron *et al*, 2003; Moebus *et al*, 2010), especially in ER-negative and PgR-negative tumours (Berry *et al*, 2006). However, benefits of dose-dense approach must be seen in the context of greater non-haematological toxic effects, myelosuppression being no longer the limiting toxicity. For this approach to be successful, it cannot jeopardise the quality of life by early acute and persistent toxicity, and feasibility still depends largely on several factors including the choice of agents, and dose and sequence (anthracyclines or taxanes first?).

More dose-dense trials have used paclitaxel (standard or new formulations) with less reported complications than with docetaxel (Citron *et al*, 2003; Lambert-Falls and Modugno, 2007; Yardley *et al*, 2008; Wildiers *et al*, 2009; Burnell *et al*, 2010; Jacot *et al*, 2010; Moebus *et al*, 2010; Robert *et al*, 2011). In the Arbeitsgemeinschaft Gynäkologische Onkologie phase III study, the rate of grade 3–4 skin toxicity observed in the intense sequential dose-dense arm (epirubicin, paclitaxel, followed by cyclophosphamide) was less than 5%, though the rates of grade 1 and 2 events reported as high as 31 and 15% cannot be ignored (Moebus *et al*, 2010).

The optimal sequence of component parts of a sequential regimen remains unclear, as published results often mix strategies (e.g., dose, length of intervals). In one study, a reverse sequence to ours was stated as feasible at least in neoadjuvant setting, yielding a high relative dose intensity and a 25% complete pathological rate; however, anthracyclines (FEC 100) were given every 3 weeks after dose-dense docetaxel at 100 mg m^−2^, and grade 3 skin toxicity was reported in one quarter of patients, questioning the real feasibility of such regimen given that the pathological complete response rate is not very different from what would be expected with conventional 3-weekly chemotherapy (Jacot *et al*, 2010). Another trial investigated a similar dose-dense reverse sequence: docetaxel followed by AC every 2 weeks. Recruitment was stopped for toxicity reasons after enrolment of 36 women, based on significant toxicity being observed in more than 50% of cases, despite systematic dose reduction applied to docetaxel from 100 to 75 mg m^−2^ (Lambert-Falls and Modugno, 2007). Of note, grade 3–4 palmar-plantar erythrodysesthesia occurred in 25% of patients during docetaxel medication *vs* none during AC, and 25% dose reduction of docetaxel did not solve the skin toxicity, grade 3 still occurring in 11% patients at the lower dosage (Lambert-Falls and Modugno, 2007), stressing that dose and sequence are not exclusive issues and may affect delivery of planned doses and toxicity (Yardley *et al*, 2008; Wildiers *et al*, 2009).

In conclusion, this phase study adds to the body of evidence that dose-dense FEC 100 followed by docetaxel 100 mg m^−2^ (FEC-D) is not feasible. Indeed the feasibility of dose-dense chemotherapy incorporating a taxane within an anthracycline-based polychemotherapy backbone remains unclear and depends on careful regimen design and a justified therapeutic ratio. Use of the reverse sequence (taxane preceding anthracycline) should be restricted to clinical studies until there is more evidence of its benefit, and any such research should also focus on defining which tumours would derive the most therapeutic benefit of using such a strategy in the place of standard scheduling.

## Figures and Tables

**Table 1:** Baseline characteristics

	**Arm A *N* (%)**	**Arm B *N* (%)**
*N*	37 (100)	37 (100)
Age, median (range), year	47 (29–64)	56 (34–67)
Conservative surgery	21 (57)	16 (43)
Mastectomy	16 (43)	21 (57)
		
*Pathology*		
Invasive lobular carcinoma	4 (11)	3 (8)
Invasive ductal carcinoma	33 (89)	33 (89)
Other	0 (0)	1 (3)
		
pT, mean (range), mm	24.0 (6–50)	28.3 (9–90)
		
*pN*+		
1–3 pN+	8 (22)	4 (11)
>3 pN+	29 (78)	33 (89)
		
*SBR grade*		
I	4 (11)	4 (11)
II	11 (30)	14 (38)
III	22 (59)	19 (51)
		
*ER and PgR status*		
ER-negative	12 (32)	12 (32)
PgR-negative	17 (46)	17 (46)
ER- and PgR-negative	11 (30)	11 (30)
ER- and/or PgR-positive	26 (70)	26 (70)
		
*HER2*		
Positive (IHC+++ or FISH+)	11 (30)	9 (24)
Negative	12 (32)	16 (43)
ND	14 (38)	13 (35)

Abbreviations: ER=estrogen receptor; FISH=fluorescence *in situ* hybridization; IHC=immunohistochemistry; *N*=number of patients; ND=not determined; PgR=progesterone receptor; pN=pathologic lymph nodes; pT=pathological size; SBR=Scarff–Bloom–Richardson.

**Table 2:** Toxicity per patient

	**Arm A (*N*=37)**			**Arm B (*N*=37)**		
	**Total**	**Cycle 1–3**	**Cycle 4–6**	**Total**	**Cycle 1–3**	**Cycle 4–6**
**Event**	***N* (%)**	***N* (%)**	***N* (%)**	***N* (%)**	***N* (%)**	***N* (%)**
*Neutropenia*						
Grade 3–4	18 (48.6)	12 (32.4)	10 (27.0)	14 (37.8)	11 (29.7)	6 (16.2)
						
Febrile neutropenia	2 (5.4)	0	2 (5.4)	1 (2.7)	1 (2.7)	0
						
*Thrombocytopenia*						
Grade 3–4	0	0	0	1 (2.7)	1 (2.7)	0
						
*Skin toxicity*						
Grade 1–2	10 (27.0)	7 (18.9)	10 (27.0)	20 (54.1)	4 (10.8)	19 (51.4)
Grade 3–4	12 (32.4)	0	12 (32.4)	7 (18.9)	0	7 (18.9)
						
*Mucositis*						
Grade 3–4	2 (5.4)	1 (2.7)	1 (2.7)	1 (2.7)	0	1 (2.7)
						
*Nail toxicity*						
Grade 1–2	7 (18.9)	0	7 (18.9)	9 (24.3)	1 (2.7)	9 (24.3)
Grade 3–4	1(2.7)	0	1 (2.7)	1 (2.7)	0	1 (2.7)
						
*Arthralgia myalgia*						
Grade 1–2	10 (27.0)	3 (8.1)	7 (18.9)	13 (35.1)	2 (5.4)	13 (35.1)
Grade 3–4	1 (2.7)	0	1 (2.7)	1 (2.7)	0	1 (2.7)
						
*Oedema*						
Grade 1–2	4 (10.8)	1 (2.7)	3 (8.1)	6 (16.2)	1 (2.7)	6 (16.2)
Grade 3–4	1 (2.7)	0	1 (2.7)	0	0	0

**Table 3:** Dose reductions and treatment delays (⩾5days)

**Cycle**	**Administration**	**Arm A (*N*=37), *N***	**Arm B (*N*=37), *N***
Cycle 1	Q2w cycles administered	37	37
	As planned	37	37
			
Cycle 2	Q2w cycles administered	35	37
	As planned	31	36
	With dose reduction	0	1
	Delayed	4	0
	Cancelled	0	0
	Q3w cycles^a^	2	NA
			
Cycle 3	Q2w cycles administered	31	37
	As planned	30	36
	With dose reduction	1	0
	Delayed	0	0
	Cancelled	0	1
	Q3w cycles^a^	6	NA
			
Cycle 4	Q2w cycles administered	23	37
	As planned	23	33
	With dose reduction	0	0
	Delayed	0	3
	Cancelled	0	1
	Q3w cycles^a^	14	NA
			
Cycle 5	Q2w cycles administered	22	37
	As planned	6	26
	With dose reduction	6	3
	Delayed	4	7
	Cancelled	6	1
	Q3w cycles^a^	15	NA
			
Cycle 6	Q2w cycles administered	20	37
	As planned	3	26
	With dose reduction	4	3
	Delayed	4	5
	Cancelled	9	3
	Q3w cycles^a^	17	NA

Abbreviation: NA=not applicable.

a Shift to a 3-week interval in remaining cycles in March 2005 and discontinuation of inclusions in arm A is seen (not applicable in arm B).
